# Correction

**Published:** 1893-03

**Authors:** W. Bryden

**Affiliations:** 26 Sudbury St., Boston


### CORRECTION.

*Journal of Comparative Medicine and Veterinary Archives:*

On page 105 of your February, 1893, issue the word *unfair* should be *unfortunate.* It occurs on the 10th line from the foot of the page.

On the 12th line from the foot of page 71 *perforatus* should be *perforans.* Yours respectfully,

W. BRYDEN.

26 SUDBURY ST., BOSTON.

